# *Helicobacter pylori* infection attenuates 2,4-dinitrochlorobenzene-induced atopic dermatitis-like skin lesions in C57/BL6 mice

**DOI:** 10.1186/s13223-023-00851-x

**Published:** 2023-11-17

**Authors:** Shuxian Wang, Xiaokang Wang, Jiaqi Liu, Yaqian Li, Minghui Sun, Guoqiang Zhu, Xiaofang Zhu

**Affiliations:** 1 Department of Dermatology, Medical College, Yangzhou University, Yangzhou, China; 2 Department of Dermatology, Clinical Medical College, Yangzhou University, Yangzhou, China; 3 Veterinary Medical College, Yangzhou University, Yangzhou, China

**Keywords:** Atopic dermatitis, *Helicobacter pylori*, Skin barrier, Pruritus, JAK–STAT

## Abstract

**Background:**

Although numerous studies have suggested a negative correlation between *Helicobacter pylori* (*H. pylori*) infection and allergies, there has been limited research on the relationship between *H. pylori* infections and atopic dermatitis (AD). The present study aimed to investigate the effects of *H. pylori* infection in an AD mouse model and identify potential mechanisms related to type 2 immunity, skin barrier defects, and pruritus.

**Methods:**

A model of AD-like symptoms was established with 2,4-dinitrochlorobenzene (DNCB) after infection of the gastric cavity with *H. pylori*. Analysis of the expression of key inflammatory cytokines and serum levels of immunoglobulin E (IgE) was based on enzyme-linked immunosorbent assay (ELISA). The expression of filaggrin (FLG) and loricrin (LOR) were analyzed by immunohistochemistry staining. The evaluation of STAT1, STAT3, phosphorylated STAT1 (phospho-STAT1), and phosphorylated STAT3 (phospho-STAT1) expression levels in skin lesions was performed using western blot.

**Results:**

The present study showed that the *H. pylori*-positive AD group (HP+AD+) exhibited milder skin lesions, including erythema, erosion, swelling, and scaling, than the *H. pylori*-negative AD group (HP−AD+). Additionally, HP+AD+ displayed lower levels of IgE in serum, and downregulated expression of interleukins 4 and 31 (IL-4 and IL-31) in serum. Furthermore, HP+AD+ demonstrated higher expression of filaggrin and loricrin than HP−AD+. Notably, *H. pylori* significantly reduced the amount of phosphorylated STAT1 and STAT3.

**Conclusion:**

*Helicobacter pylori* infection negatively regulates the inflammatory response by affecting inflammatory factors in the immune response, and repairs the defective epidermal barrier function. In addition, *H. pylori* infection may reduce IL-31, thereby alleviating pruritus. These effects may be associated with the inhibition of JAK–STAT signaling activation.

**Supplementary Information:**

The online version contains supplementary material available at 10.1186/s13223-023-00851-x.

## Background

Globally, atopic dermatitis (AD) affects approximately 30% of infants, 10%–20% of children, and 1%–3% of adults [1]. A wide variety of clinical manifestations are associated with the disorder, including intense pruritus and symptoms of recurrent eczema [2]. AD patients often develop other allergic diseases. The allergic march, also known as the atopic triad, begins with AD and may later involve food allergy, asthma, and allergic rhinitis. Chronic and relapsing AD poses significant social and economic burdens with increased indirect costs, including medical appointments, work or school absenteeism, and hospitalizations [3]. Although the pathogenesis of AD is still unclear, a large body of evidence suggests that several factors play a role here, including genetics (filaggrin protein mutations), environmental factors (allergens), and immune dysfunction [2].

Around 4.4 billion people worldwide are afflicted with the gram-negative bacterium *Helicobacter pylori* (*H. pylori*) [4]. *H. pylori* infects both adults and children [4, 5] and may cause chronic gastritis, peptic ulcer, and gastric cancer. The severity of the diseases caused by *H. pylori* is influenced by its virulence factors, which have been identified as cytotoxin-associated antigen A (CagA), vacuolating cytotoxin (VacA), duodenal ulcer-promoting gene A protein (DupA), and gamma-glutamyl transpeptidase (GGT) [4]. Exposure to *H. pylori* may also lead to immune tolerance and reduced risk of atopic diseases [5].

The JAK–STAT pathway modulates multiple immune pathways central to the immunopathogenesis of AD. Specifically, it governs the signaling of cytokines such as interleukins 4 and 31 (IL-4 and IL-31), which contribute to the chronic inflammation and pruritus symptoms in AD. Additionally, the JAK–STAT pathway regulates the epidermal barrier and influences peripheral nerves associated with pruritus transmission [6].

According to the “hygiene hypothesis,” the improvement of hygiene conditions and the gradual loss of local microbial communities lead to a decrease in early childhood infections or microbial exposure, which in turn promotes the development of allergic diseases [7]. However, recent studies have suggested that the hygiene hypothesis does not only involve exposure to microorganisms but may also be related to the development and function of regulatory T cells (Tregs), a type of immune cell that can suppress excessive immune responses and maintain immune balance [8]. It has been suggested that developing and developed countries show an increased risk of inflammatory diseases such as allergies, asthma, and autoimmune disorders in later life due to a decrease in immunomodulatory microorganisms such as *H. pylori* [9, 10]. Specifically, chronic *H. pylori* infection occurs rarely in individuals with asthma, hay fever, or eczema, especially in children [11]. Hence, *H. pylori* infection may decrease the risk of AD.

2,4-Dinitrochlorobenzene (DNCB), as an irritant agent, possesses the capability to incite allergic contact dermatitis (ACD). Given the intricate and multifaceted factors intertwined with the pathogenesis of AD, the practice of epicutaneous sensitization with DNCB stands as a commonly employed methodology to emulate symptoms and molecular mechanisms akin to those observed in AD [12, 13].

Although many studies have indicated a negative correlation between *H. pylori* infection and allergies and asthma [14, 15], few studies have focused on the relationship between AD and *H. pylori* infection [1, 16] and the available results are contradictory [16, 17]. We hypothesized the existence of a negative correlation between *H. pylori* infection and AD symptoms. Therefore, we used DNCB-treated C57/BL6 mice to detect the effect of *H. pylori* infection on AD-like symptoms and demonstrate its mechanisms related to type 2 immunity, skin barrier defects, and pruritus.

## Methods

### Animals and bacteria

Six-week-old C57/BL6 female mice (from the Experimental Animal Center of Yangzhou University, Yangzhou, China) were used in this experiment. They were fed solid food (without antibiotics) and given free access to water. The animals were acclimated for a week at 24 ± 1 °C and a 12-h normal light-and-dark cycle before the experiment. The animal experiments were approved by the Ethics Committee for Experimental Animal Research of Yangzhou University. Additional file 1 is the ethics approval document.

*H. pylori* SS1 (gift from Nanjing University) was cultured on Colombia Blood Agar Base (Thermo Scientific, Waltham, USA) containing 5 μg/mL trimethoprim, 2 μg/mL amphotericin B, 10 μg/mL vancomycin, and 3.8 μg/mL polymyxin. For mouse infection, *H. pylori* were grown under shaking in brain heart infusion (BD, Franklin Lakes, USA). Then, the cultures were incubated in an anaerobic incubator (Hua Yue, Guangzhou, China) at 37 °C with 5% O_2_, 10% CO_2_, and 85% N_2_ for 4 days.

### *H. pylori* infection model

After fasting overnight, the mice were gavaged once per day for five consecutive days with 300 μL phosphate-buffered solution (PBS) containing 1 × 10^9^ CFU/mL *H. pylori*. The control mice were inoculated with saline solution.

### AD-like skin lesions mouse model

Several concentrations of DNCB (Sigma-Aldrich, St. Louis, MO, USA) were used to induce AD-like lesions of the skin after 6 weeks of infection with *H. pylori*. In the first stage of inflammation induction, the control group (HP−AD−) was treated only with a carrier (acetone: olive oil = 4:1), whereas in the *H. pylori*-negative AD group (HP−AD+) and the *H. pylori*-positive AD group (HP+AD+), 200 μL of 1% DNCB solution was applied to the same back area two times over the course of one week. Following the sensitization, 100 μL of 0.5% DNCB solution was repeatedly applied on the back skin twice a week for 14 days.

### Measurement of dermatitis score

The severity of AD-like skin lesions was measured by scoring as described previously [18]. Each of the four symptoms (erythema/hemorrhage, edema, excoriation/erosion, and scaling/dryness) was graded as follows: 0 (none), 1 (mild), 2 (moderate), or 3 (severe). The sum of all individual scores for the four symptoms of AD-like changes was calculated (maximum score: 12).

### Scratching behavior of mice

On the 21st day, after stimulation with DNCB, the mice were placed separately, and we counted how many times in a 60-min period the mice scratched their backs or brushed against the cage. Continuous scratching was counted as one time, and the number of scratches was counted.

### Histological examination

Tissue slides were made as previously reported [15]. After hematoxylin and eosin (H&E) and toluidine blue staining, anti-filaggrin (Signal, College Park, USA) and anti-loricrin (Abcam, Cambridge, UK) antibodies were used for immunohistochemistry. The Ultravision Quanto detection system (Thermo Fisher Scientific, MA, USA) was employed for the staining. Following the staining process, the tissue was dehydrated and sealed in a fixed culture medium (Sinopharm Group; Shanghai, China), and it was examined under an optical microscope (Olympus, Tokyo, Japan).

### Quantification of total serum inflammatory cytokines and immunoglobulin E (IgE)

Blood samples were collected from the mice and centrifuged at 4 °C for 30 min. Enzyme-linked immunosorbent assay (ELISA) kits (ELISA LAB, Wuhan, China) were employed for the measurement of serum IgE, IL-4, and IL-31 levels, following the guidelines provided by the manufacturer. We measured the absorbance at 450 nm using a microplate reader (BioTek Epoch, Vermont, USA).

### Western blot analysis

After homogenizing the back tissue in radioimmunoprecipitation assay (RIPA) lysis buffer (Beyotime, Shanghai, China) and supplementing it with protease and phosphatase inhibitor cocktails (Beyotime, Shanghai, China), a bicinchoninic acid (BCA) protein assay kit (Beyotime, Shanghai, China) was used to determine the levels of protein in the lysates. Subsequently, equivalent protein quantities were loaded and separated on 10% sodium dodecyl sulfate–polyacrylamide gel electrophoresis (SDS-PAGE) gels, followed by transfer onto polyvinylidene fluoride membranes (Vazyme, Nanjing, China). The membranes were treated with primary antibodies against anti-phospho-STAT1, anti-STAT1, anti-phospho-STAT3, and anti-STAT3 (Cell Signaling Technology, MA, USA) as well as anti-actin (Santa Cruz Biotechnology, CA, USA) at 4 °C overnight after being blocked with QuickBlockTM blocking buffer (Beyotime, Shanghai, China). Following the washing step, the membranes were subjected to incubation with secondary antibodies (anti-mouse or anti-rabbit from Cell Signaling Technology) conjugated to horseradish peroxidase (HRP). Finally, using enhanced chemiluminescence tools (ECL, NCM Biotech, Suzhou, China), immunoreactive signals were detected.

### Statistical analysis

Data were analyzed and plotted through GraphPad Prism 5 software. Continuous variables were represented by the mean ± standard error of mean (SEM), while counts and percentage values were used to represent discrete variables. Student *t*-tests or one-way analysis of variance (ANOVA) were performed to compare values between groups, as appropriate. Post hoc tests were performed using Tukey’s honestly significant difference (HSD) method to identify significant differences among multiple groups. P < 0.05 was considered statistically significant.

## Results

### *DNCB-induced AD-like lesions are less severe in HP*+*AD*+

**Fig. 1:**
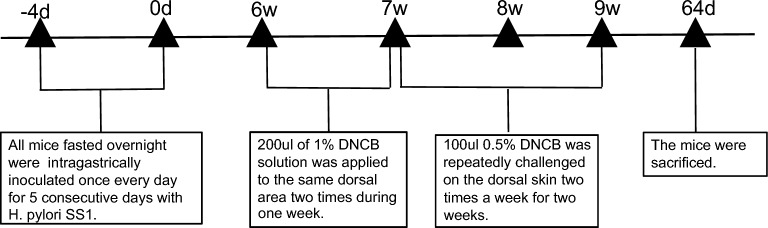
Procedure used in the experiments to establish atopic dermatitis (AD) and *Helicobacter pylori* (*H. pylori*) infection in C57BL/6 mice. Over a period of five days, the mice were intragastrically inoculated with 300 mL phosphate-buffered saline containing 1 × 10^9^ CFU/mL *H. pylori* SS1 after overnight fasting. Age-matched control mice were given physiological saline devoid of *H. pylori* SS1. After 6 weeks of *H. pylori* infection, 2,4-dinitrochlorobenzene (DNCB) was repeatedly applied to a local site to produce skin lesions that resembled AD. All groups, with the exception of the control group (HP-AD-group), received 200 µL of 1% DNCB solution twice daily for a week as part of the initial inflammation induction process. The HP-AD- group received treatment using the vehicle. Following the sensitization, the dorsal skin was repeatedly challenged with 100 µL of 0.5% DNCB dissolved in acetone and olive oil (4:1) twice a week for two weeks

**Fig. 2:**
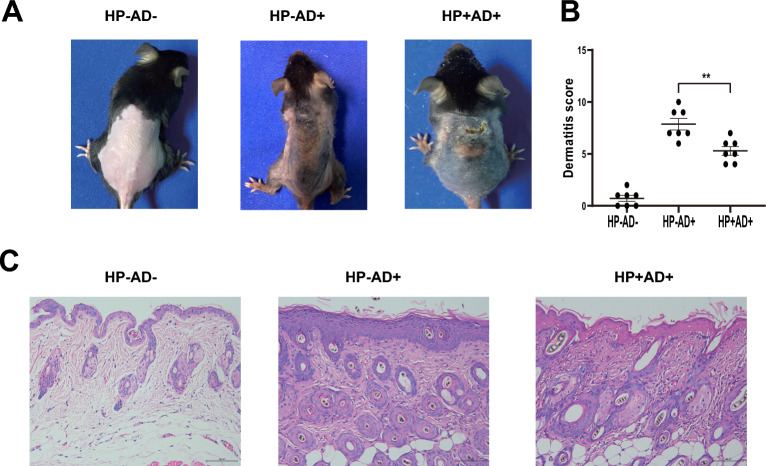
Severity of skin lesions in all of the experimental groups (**A**). Skin lesions in the *H. pylori*-negative AD group (HP−AD+) and the *H. pylori*-positive AD group (HP+AD+). **B** Dermatitis score in the control (HP−AD−), *H. pylori*-negative AD, and *H. pylori*-positive AD groups. **C** H&E staining of skin sections (200 ×) in the control group, *H. pylori*-negative AD group, and *H. pylori*-positive AD group. The representative data are shown from at least three independent experiments. Data are presented as the mean ± SEM from seven mice per group (*P < 0.05; **P < 0.01)

By gavaging mice with *H. pylori* and applying DNCB on the back skin, a mouse model to concurrently simulate both *H. pylori* infection and AD was successfully established (Fig. 1). After 21 days of DNCB application, the severity of skin lesions in the HP+AD+ group was milder than that in the HP−AD+ group (Fig. 2A, B).

**Fig. 3:**
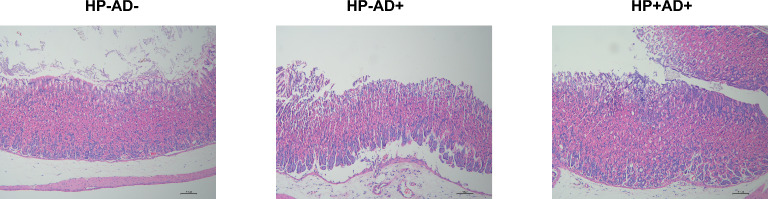
H&E staining of gastric mucosal tissues in all of the experimental groups. H&E staining of gastric mucosal tissues (100×) from the control group (HP−AD−), *H. pylori*-negative AD group (HP−AD+), and *H. pylori*-positive AD group (HP+AD+)

Histopathological examination of the back skin revealed prominent features of epidermal hyperplasia, infiltration by inflammatory cells, and intracellular edema in the AD mice induced by DNCB. HP−AD+ showed more significant epidermal hyperplasia and inflammatory cell infiltration than HP+AD+ (Fig. 2C). Histological examination of a sample of gastric mucosal tissue removed from a dead mouse demonstrated the existence of *H. pylori* in HP+AD+ (Fig. 3).

**Fig. 4:**
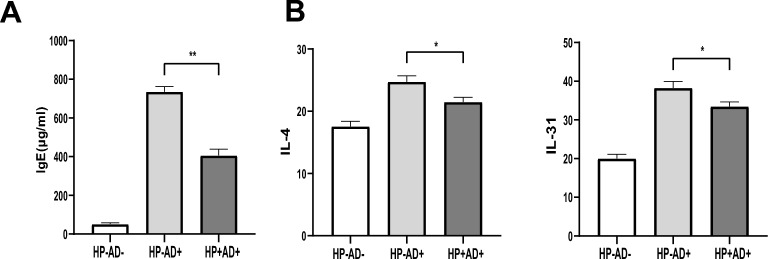
Effect of *H. pylori* on immunoglobulin E (IgE), interleukin-4 (IL-4), and IL-31 expression in all of the experimental groups (**A**) Levels of serum IgE. Levels of serum IgE in the control group (HP−AD−), *H. pylori*-negative AD group (HP−AD+), and *H. pylori*-positive AD group (HP+AD+) were examined by ELISA. **B** Expression of inflammatory cytokines in serum samples. The expression of IL-4 and IL-31 in serum was examined by ELISA. The representative data are shown from at least three independent experiments. Data are presented as the mean ± SEM from seven mice per group (*P < 0.05; **P < 0.01)

The measurement of serum IgE levels was performed using ELISA. The serum IgE levels in HP−AD−, HP−AD+, and HP+AD+ were 50.85 ± 6.70 μg/mL, 735.01 ± 27.17 μg/mL, and 406.10 ± 32.44 μg/mL, respectively. In HP+AD+, the serum IgE level was considerably lower than that in HP−AD+ (P < 0.01) (Fig. 4A).

### Expression of inflammatory cytokines in serum

The levels of inflammatory cytokines IL-4 and IL-31 in HP−AD+ and HP+AD+ groups were higher than those in HP−AD−. As shown in Fig. 4B, the expression of IL-4 (P < 0.05) and IL-31 (P < 0.05) in HP+AD+ was significantly lower than that in HP−AD+. These findings indicate that *H. pylori* can suppress the immune response in AD by regulating the level of type 2 immunity–related cytokines.

### *H. pylori* contributes to the restoration of the skin barrier in mice with DNCB-induced AD

**Fig. 5:**
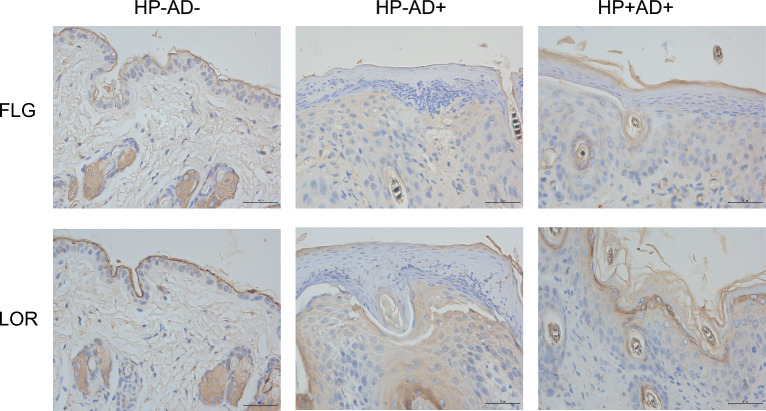
Expression of skin barrier proteins in all of the experimental groups. Expression of filaggrin (FLG) and loricrin (LOR) as evaluated by immunohistochemical staining in the control group (HP−AD−), *H. pylori*-negative AD group (HP−AD+), and *H. pylori*-positive AD group (HP+AD+)

We investigated the potential of *H. pylori* to ameliorate AD-like skin lesions through its impact on the changes in their expression of epidermal differentiation proteins, specifically filaggrin (FLG) and loricrin (LOR), to assess alterations in skin barrier function. We found that the expression of FLG and LOR in DNCB-induced AD-like skin lesions was significantly reduced, compared with HP−AD− (Fig. 5). However, the expression level was higher in HP+AD+ than in HP−AD+ (Fig. 5). These results suggest that *H. pylori* may be effective in accelerating skin barrier repair.

### *H. pylori* inhibits scratching and suppresses phosphorylated STAT in AD mice induced by DNCB

**Fig. 6:**
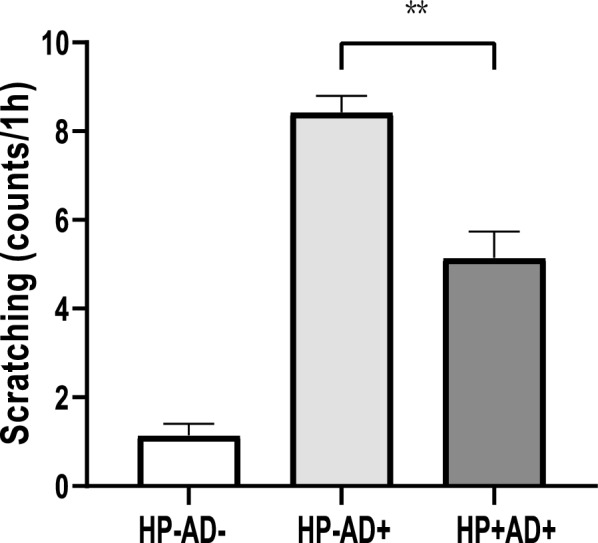
Number of scratches in all of the experimental groups. Number of scratches in 60 min on the 21st day after DNCB application in the control group (HP−AD−), *H. pylori*-negative AD group (HP−AD+), and *H. pylori*-positive AD group (HP+AD+). The representative data are shown from at least three independent experiments. Data are presented as the mean ± SEM from seven mice per group (*P < 0.05; **P < 0.01)

**Fig. 7:**
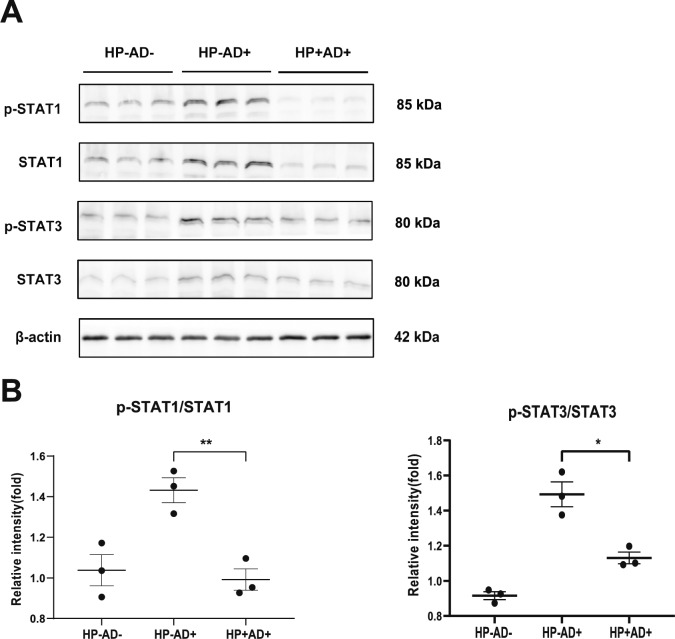
Expression of anti-phosphorylated-STAT1 (anti-p-STAT1), anti-STAT1, anti-phosphorylated-STAT3 (anti-p-STAT3), and anti-STAT3 in all of the experimental groups. **A**, **B** Immunoblots with anti-p-STAT1, anti-STAT1, anti-p-STAT3, anti-STAT3, or anti-β-actin antibodies using lysates from the control group (HP−AD−), *H. pylori*-negative AD group (HP−AD+), and *H. pylori*-positive AD group (HP+AD+). Western blots were analyzed quantitatively. The representative data are shown from at least three independent experiments. Data are presented as the mean ± SEM from seven mice per group (*P < 0.05; **P < 0.01)

To assess the degree of pruritus, on the 21st day of DNCB treatment, the frequency with which the mice scratched their backs or rubbed against their cages within 60 min after DNCB stimulation was recorded and quantified. Compared with HP−AD+, HP+AD+ had a reduced number of scratches (Fig. 6, P < 0.01). Our results showed that *H. pylori* significantly reduced pruritus and suppressed IL-31 (Fig. 4B, P < 0.05). Furthermore, we investigated the effect of *H. pylori* on JAK-STAT signaling in AD-like lesions by evaluating the activation of STAT1 and STAT3 in the dorsal skin. Our findings demonstrated that *H. pylori* infection significantly decreased phosphorylated STAT1 and STAT3 levels that were induced by DNCB (Fig. 7A, B). Therefore, our results indicate that *H. pylori* infection improves AD by suppressing STAT1 and STAT3 signaling, which are involved in the expression of type 2 immunity–related cytokines.

## Discussion

Recent studies have suggested that the ability of *H. pylori* to modulate the immune system may be beneficial in treating allergic diseases, including asthma and dermatitis [1, 19, 20]. Interestingly, *H. pylori* eradication therapy with antibiotics after the sensitization phase has been shown to result in the efficient killing of the bacteria and the disappearance of the protective effect against asthma [21]. However, another study reported that *H. pylori* infection did not confer protection against atopy [5].

According to the hygiene hypothesis, *H. pylori* infection can lead to substantial immune tolerance, resulting in a lower occurrence of atopic and allergic diseases, which could explain their negative correlation [22]. A recent study has explained the molecular mechanism underlying the hygiene hypothesis associated with *H. pylori* infection, reporting that it involves a larger Treg response that is mediated by dendritic cells [23]. However, the reason why chronic microbial infections can inhibit type 2 immunity cell-mediated autoimmune diseases involves IL-10–dependent counter-regulation [24]. Daniela et al. have shown that dendritic cells can produce IL-10 to suppress lung allergen-specific immune responses, while Tregs are not necessary for the protective effects mediated by *H. pylori* extract [11]. However, there have been few studies on the relationship between AD and *H. pylori* [1, 16].

There is some evidence that the microbial influence on allergic diseases is typically regulated by genetic mechanisms. Microorganisms modulate immune responses, including the production of cytokines such as IL-6 and IL-10, to affect the development of immune reactions and protection against allergic diseases [25, 26]. These gene–environment interactions may shape epigenetic changes within immune cells, ultimately affecting the susceptibility of individuals to allergies [27]. It is worth considering whether similar microbial effects are also present in other allergic conditions. For example, the potential role of *H. pylori* in the development of AD could also involve gene regulation, thereby adding another dimension to the interplay of genetics, environment, and epigenetics in allergic diseases.

The causes of AD include T-lymphocyte polarization, mast-cell proliferation, and a high level of IgE [28]. Typically, the immune response begins with the activation of type 2 immunity cells, eventually progressing into a chronic disease state characterized by a mixed type 2 immunity/type 1 immunity cytokine profile. Specifically, in acute skin lesions of AD, type 2 immunity cells play a significant role in producing cytokines such as IL-4 and IL-13. As the disease progresses, the type 2 immunity reaction increases with the increase in Th1 reaction biomarkers. In addition, IL-4 plays a crucial role in the proliferation and differentiation of B cells, leading to the generation of IgE antibodies by stimulating eosinophils in the inflammatory area [28]. IL-31, released by type 2 immune cells, can induce itch by directly stimulating IL-31 receptor subunit α (IL-31Rα) or by interacting with neurons that express transient receptor potential vanilloid 1 and ankyrin 1 (TRPV1) or transient receptor potential ankyrin 1 (TRPA1) receptors [2]. Epithelial damage caused by scratching triggers innate immune activation, which is characterized by the release of proinflammatory cytokines by keratinocytes. Additionally, skin-resident dermal dendritic cells participate in antigen-presentation processes [29]. In our study, the mice were intragastrically inoculated with *H. pylori* SS1 every day for a total of five times following an overnight fasting. Six weeks after the *H. pylori* infection, we repeatedly applied DNCB locally to induce AD-like skin lesions. We found an increase in IL-4 and IL-31 serum levels in the mice treated with DNCB relative to HP−AD−. As expected, the levels of IL-4 and IL-31 were reduced in the group subjected to *H. pylori* gastric lavage (Fig. 4B). Furthermore, the presence of *H. pylori* in the stomach markedly diminished the increase in serum level of IgE, which is a marker of AD (Fig. 4A). These results indicate that *H. pylori* infection inhibits DNCB-induced skin inflammation in AD-like mice by regulating the inflammatory environment.

AD is a skin disorder with both compromised skin barrier function and persistent itching symptoms, which is associated with elevated levels of IL-4 and IL-13. A decrease in the concentrations of molecules associated with the skin barrier, such as FLG and LOR (which are important for the structure and function of the epidermis), is linked to impaired skin barrier function. The increase in IL-4 and IL-13 can strongly reduce the levels of these molecules [30]. In the present study, we verified the diminished expression of FLG and LOR in the AD region in mice (Fig. 5), but *H. pylori* infection was able to reverse the downregulation of the barrier-related proteins. In addition, *H. pylori* significantly inhibited the activation of STAT1 and STAT3 in the AD-like region (Fig. 7A, B). The exact mechanism and relevant target cell types of *H. pylori* and the determining factors of *H. pylori* in vivo remain to be elucidated in detail.

Although *H. pylori* has notable immunomodulatory properties and is inversely associated with various allergic diseases, its use as a therapeutic strategy or defensive action is unappealing because of its existing potential to cause cancer through chronic infection. Gastroduodenal ulcers also brought on by *H. pylori* are one of the main factors in the development of carcinoma [11]. However, *H. pylori* extract or its immunomodulator vacuolating cytotoxin exposure during pregnancy can offer significant protection from inflammatory airway allergies, not only for the first generation but also for subsequent generations, without increasing vulnerability to bacterial and viral infections [9]. Several studies have indicated that *H. pylori* infection has an inverse relationship with allergic rhinitis and asthma, especially in young people with diseases with an early onset. Compared with children, adolescents, and young people, elderly individuals benefit the least from having *H. pylori* in their stomach [31, 32]. A study suggested that the two determining factors for the persistence of *H. pylori* in preventing asthma are GGT and VacA, which can be purified to prevent asthma [11].

## Conclusions

In summary, this study suggests a negative correlation between *H. pylori* infection and AD-like inflammation, which may be associated with the regulation of inflammatory response and the repair of defective epidermal permeability barrier function. Additionally, *H. pylori* reduces pruritus by inhibiting JAK–STAT signaling activation and decreasing IL-31 levels. However, a limitation of this study is that only seven mice were used in each group, indicating that the power of the study was limited. To prevent AD while avoiding the risks of *H. pylori* infection, a strategy of *H. pylori*–specific tolerization that harnesses the immunomodulatory properties of bacteria during early childhood needs to be developed. More studies are required to clarify several important factors that negatively impact the relationship between AD and *H. pylori*.

### Supplementary Information

**Additional file 1.** Ethics approval document

## Data Availability

The datasets used and/or analyzed during the current study are available from the corresponding author on reasonable request.

## Abbreviations

*H. pylori*: *Helicobacter pylori*
AD: Atopic dermatitis
DNCB: 2,4-Dinitrochlorobenzene; allergic contact dermatitis (ACD)
IgE: Immunoglobulin E
ELISA: Enzyme-linked immunosorbent assay
phospho-STAT1: Phosphorylated-STAT1
phospho-STAT3: Phosphorylated-STAT3
HP+AD+: *H. pylori*-Positive AD group
HP−AD+: *H. pylori*-Negative AD group
HP−AD−: The control group
IL-4: Interleukin-4
IL-31: Interleukin-31
CagA: Cytotoxin associated antigen A
VacA: Vacuolating cytotoxin
DupA: Duodenal ulcer promoting gene A protein
GGT: Gamma-glutamyl transpeptidase
Tregs: Regulatory T cells
PBS: Phosphate buffer solution
H&E: Hematoxylin and Eosin
RIPA: Radioimmunoprecipitation assay
BCA: Bicinchoninic acid
SDS-PAGE: Sodium dodecyl sulfate–polyacrylamide gel electrophoresis
HRP: Horseradish peroxidase
ECL: Enhanced chemiluminescent
SEM: Standard error of mean
ANOVA: Analysis of variance
HSD: Honestly significant difference
IL-31Rα: IL-31 receptor subunit α
TRPV1: Transient receptor potential vanilloid 1 and ankyrin 1
TRPA1: Transient receptor potential ankyrin 1
