# Supplementary material for: Helicobacter pylori infection attenuates 2,4-dinitrochlorobenzene-induced atopic dermatitis-like skin lesions in C57/BL6 mice
Source: Allergy Asthma Clin Immunol. 2023 Nov 17;19:97. doi: 10.1186/s13223-023-00851-x (PMC10656826; doi:10.1186/s13223-023-00851-x)
Supplement: Supplementary file 1 — Additional file 1. Ethics approval document [file 13223_2023_851_MOESM1_ESM.pdf]

扬州大学实验动物福利伦理审查表

编号（No）：　　　　　　　　　　　　　　　　　　　日期：2022-03-02

<table>
<tr><td colspan="2">申请单位</td><td colspan="3">兽医学院(比较医学研究院)</td></tr>
<tr><td colspan="2">申请人</td><td>朱国强</td><td>联系电话</td><td>13773564198</td></tr>
<tr><td colspan="2">负责人</td><td>朱国强</td><td>联系电话</td><td>13773564198</td></tr>
<tr><td colspan="2">课题负责人邮箱</td><td colspan="3">yzgqzhu@yzu.edu.cn</td></tr>
<tr><td colspan="2">实验名称</td><td colspan="3">微量快速特异敏感性幽门螺杆菌感染性抗体定性定量检测方法和临床应用研究</td></tr>
<tr><td colspan="2">课题级别</td><td colspan="3">省部级</td></tr>
<tr><td colspan="2">实验开始日期</td><td>2022-01-01</td><td>结束日期</td><td>2025-12-31</td></tr>
<tr><td rowspan="4">拟进动物情况</td><td>动物来源</td><td colspan="3">1. 济南斯帕法斯家禽有限公司（鸡）2. 扬州大学（小鼠）</td></tr>
<tr><td colspan="2">动物实验设施合格证编号</td><td colspan="2">1.SCXK(鲁)2018-0005(鸡) 2. SCXK(苏)2017-0007(小鼠)</td></tr>
<tr><td>品种品系</td><td>来航鸡、BALB/c 小鼠</td><td>等级</td><td>SPF 级</td></tr>
<tr><td>规格</td><td>1. 来航鸡，5-7 日龄，雌雄均可 2. BALB/c 小鼠，6 周龄雌性 3. 2. BALB/c 小鼠，6 周龄雄性</td><td>数量</td><td>共 220 只</td></tr>
<tr><td>饲养设施条件</td><td colspan="4">屏障设施，本设施的环境条件符合中国国家标准《实验动物环境及设施》（GB14925-2001）对屏障动物实验设施的有关标准，动物饲养管理和动物实验操作符合相关法规的要求。</td></tr>
</table>

<table>
<tr><td rowspan="3"></td><td colspan="3">普通设施，本设施的环境条件符合中国国家标准《实验动物环境及设施》（GB14925-2001）对普通动物实验设施的有关标准，动物饲养管理和动物实验操作符合相关法规的要求。</td></tr>
<tr><td rowspan="2">设施许可</td><td>许可证编号:
SYXK(苏)2021-0027</td><td>有效期:
二０二一年三月二十六日至二０二六年三月二十五日</td></tr>
<tr><td colspan="2">许可证发放机构：江苏省科技厅</td></tr>
<tr><td colspan="4">实验要点，包括实验目的、实验方法、观测指标、实验结束后处死动物的方法等。
实验目的:
建立微量快速特异敏感性幽门螺杆菌感染性抗体定性定量检测方法和临床应用研究
实验方法:
选取 5-7 日龄 SPF 鸡，以三种不同剂量感染幽门螺杆菌，经口服和注射途径感染。

选取 6 周龄 BALB/c 小鼠，以三种不同剂量感染幽门螺杆菌，经口服和注射途径感染。

观测指标:
SPF 鸡和普通 BALB/c 小鼠:记录临床症状和发病率，于攻毒后第 1、3、5、7、14 和 21 周分别对 2 只鸡/小鼠实施安乐死，采集血液、心、肝、脾、肺、肾、脑、各肠段等器官，分离血清和分离培养和检测组织细菌载量;凝集试验检测病菌感染抗体，同时采用 ELISA 和 Western blot 检测病菌感染抗体。
实验结束后处死动物的方法:
1.实验动物处死方法:实验结束对存活动物先用舒泰麻醉后再用二氧化碳窒息法实施安乐死。
2.尸体处理方法:对动物体进行无害化处理，具体方法是，先经高温高压灭菌消毒，再妥善密封包装，然后交至扬州大学动物尸体处理中心集中处理。
3.多余动物处理方法:无多余试验动物。

其他说明：无</td></tr>
<tr><td>审
查
依
据</td><td colspan="3">1.该项目是否必须用实验动物进行实验，即能否用计算机模拟、细胞培养等非生命方法替代动物或用低等动物替代高等动物进行实验;
2.表中所填申请人资格和所用动物的品种品系、质量等级、规格是否合适，能否通过改良设计方案或用高质量的动物来减少所用动物的数量;
3.能否通过改进实验方法、调整实验观测指标、改良处死动物的方法，来优化实验方案、善待动物。</td></tr>
<tr><td colspan="4">我将自觉遵守实验动物福利伦理原则，随时接受实验动物福利与伦理委员会的监督与检查，如违反规定，自愿接受处罚。</td></tr>
</table>

声明人签名：朱国强<br>日期：2022-03-02
课题负责人意见：<br>同意<br>签字：朱国强<br>2022-03-02
实验动物福利伦理委员会审议结果：<br>经实验动物福利伦理委员会审议，授权审核。<br>签字：
填表说明：<br>要写明项目的意义、必要性、项目中有关实验动物的用途、饲养管理或实验处置方法、预期出现的对动物的伤害、处死动物的方法、项目进行中涉及实验动物伦理问题的详细描述。
